# Proteomic study of cerebrospinal fluid in patients with vascular cognitive impairment‐no dementia

**DOI:** 10.1002/alz.089147

**Published:** 2025-01-09

**Authors:** Sisi Peng, Junjian Zhang

**Affiliations:** ^1^ Zhongnan Hospital of Wuhan University, Wuhan, Hubei China; ^2^ Zhongnan Hospital, Wuhan University, Wuhan China

## Abstract

**Background:**

Vascular cognitive impairment (VCI), including vascular cognitive impairment‐no dementia (VCIND) and vascular dementia, is a cognitive impairment syndrome caused by cerebrovascular disease and its risk factors. Among people over 60 years old, the prevalence of VCIND is about 15‐20%. VCIND, as the early stage of VCI, has become a focus of current research due to the fact that its patients are at greater risk of developing dementia and can benefit greatly from early intervention. The etiology and pathology of VCI are highly heterogeneous. Analyzing the key molecules in the development of VCI through omics approaches is conducive to a multidimensional understanding of the disease nature of VCI, and is important for identifying its targets for intervention.

**Method:**

We screened for differential proteins in the cerebrospinal fluid (CSF) of VCIND patients and healthy control subjects by proteomic assays based on ultra‐performance liquid chromatography‐mass spectrometry (UPLC‐MS). Differential proteins and scores of cognitive assessments were subjected to pearson correlation analysis to screen out the differential proteins with correlation coefficients greater than 0.75. We constructed a mouse model of bilateral carotid artery stenosis (BCAS) and a cellular model of oxygen glucose deprivation (OGD), and targeted the differential proteins screened in CSF in the classic animal and cellular models of VCI mentioned above.

**Result:**

We found significant between‐group differences in MoCA (p<0.001) and DSST (p<0.001) scores in non‐demented VCI patients compared to healthy controls. 75 differential proteins were screened in the cerebrospinal fluid of non‐demented VCI patients (fold change = 1.5, p<0.05), of which 38 were up‐regulated and 37 were down‐regulated. In cerebrospinal fluid, Cerebellin‐2 (Cbln2) was the only protein with a correlation over 0.75 for both MoCA and DSST assessment, and was significantly negatively correlated with cognitive assessments. Cbln2 expression was significantly higher in hippocampal tissues of BCAS mice as well as in OGD‐treated neuronal cells, which follows the same trend as the clinical findings.

**Conclusion:**

Cbln2 is a potentially important molecule in early stage of VCI.

